# Our Warriors' Dependants and "Relief"

**Published:** 1914-09-19

**Authors:** J. C. Pringle

**Affiliations:** Acting Secretary, Charity Organisation Society. Denison House, Vauxhall Bridge Road, London, S.W.


### THE EDITOR'S LETTER-BOX.

### Our Warriors' Dependants and "Relief."

*To the Editor of* THE HOSPITAL.

SIR,—I observe in column two of page 641 of your issue of September 12 that you say that the dependants of men who are fighting our battles have gone hungry already, and that they have been passed on to the Charity Organisation Society, and thence to the Poor-Law Guardians.

As you are aware, the business of relieving the dependants of men engaged in the national defence has been entrusted to the Soldiers' and Sailors' Families' Association. Consequently, if these dependants have gone hungry, your quarrel lies rather with that body than with this Society or the Poor-Law Guardians.

It is no business of mine to attack the Association, nor is it the fault of the Association that Germany rushed Europe into a great war in such haste. When the emergency arose the representatives of the Association in many parts of London sought the co-operation of people interested and accustomed to the relief of distress. A great many people have been working in consequence seven days a week and very long hours ever since the outbreak of war, endeavouring to secure that dependants of sailors and soldiers shall be sympathetically treated and adequately relieved. We are in a position to substantiate the claim that, but for the efforts of our members, these people might have gone hungry owing to the huge and sudden rush of work with which the Soldiers' and Sailors' Families' Association found itself confronted.

Where anyone who has had an opportunity of working in the interests of these dependants has shirked that duty, I agree with you that it is a matter for indignation, but it is not a charge you will be able to substantiate against this Society. I believe that where the Poor-Law Guardians were relieving cases they have continued to do so, to secure that the cases were adequately relieved.—Yours truly, J. C. PRINGLE, Acting Secretary, Charity Organisation Society.

Denison House, Vauxhall Bridge Road, London, S.W., September 11, 1914.

[The particular case we had in mind was that of a widow in Ashmill Street, Marylebone, particulars of whose case were given in the *Marylebone Record* of August 29, and referred to in the *Daily Herald* of September 3. We brought no charge of any kind against Mr. Pringle's Society. In the circumstances under discussion the Society is a mere incident, and ought never to have been troubled in the matter at all. The C.O.S. has done, and is doing, no doubt, excellent work, but the maintenance of the dependants of our sailors and soldiers is the work of the nation, not of this Society or any other organisation which has dealt, or was constituted to deal, with distress or relief. No doubt the war came with a rush. Mistakes have resulted, but those mistakes must be ended forthwith. Neither the Poor Law nor the Charity Organisation Society nor any Charity must be made the channel through which the provision for the dependants of our sailors and soldiers is paid by the Government. Mr. Asquith's great speech at the Guildhall struck the right note, and it would indeed be an outrage to treat these dependants of our sailors and soldiers as persons in distress or objects of charity. If they were either, the nation would fail in its duty, and deservedly win the contempt of all civilised peoples throughout the world. An article on this subject appears on another page.—ED. THE HOSPITAL.]

